# Vitreous Humour Extrusion after Suxamethonium Induction of Anaesthesia in a Polytraumatized Patient: A Case Report

**DOI:** 10.1155/2010/913763

**Published:** 2010-12-27

**Authors:** Frederick Ebegue Amadasun, Theodore Ojeide Isesele

**Affiliations:** Department of Anaesthesiology, University of Benin Teaching Hospital, PMB 1111, Benin City, Nigeria

## Abstract

*Introduction*. Suxamethonium, a deepolarizing muscle relaxant, increases intraocular pressure. It is therefore advised to be avoided in open globe surgery, for fear of extruding ocular contents. Several anecdotal reports support this fear. Some workers however, dispute this claim. There is as yet no formal case report in the literature on the subject. *Case Presentation*. A 34-year old Nigerian male, was involved in a road traffic accident. He presented at the Accident & Emergency Unit of our hospital about 2 hours after the accident. Clinical examination revealed right corneal laceration (with intact ocular contents) and intra-abdominal visceral injury. Emergency laparotomy was scheduled, to be followed with corneal repair. Anaesthesia was induced with 10 mg midazolam, 100 mg ketamine, and 100 mg suxamethonium given intravenously in sequence. After laparotomy, the ophthalmologists reported for the corneal repair, only to find that the vitreous humour has been extruded. *Conclusion*. The fear about the use of suxamethonium in open globe situations is real. It will be good clinical judgment to use alternative drugs and techniques to effect rapid muscle relaxation, in the anaesthetic management of the open globe patient. This would be of interest to anaesthetists, ophthalmologists and clinical pharmacologists among others.

## 1. Introduction

Suxamethonium, a depolarizing muscle relaxant, is frequently used by anaesthetists to produce fast-onset muscle relaxation for endotracheal intubation. Part of its pharmacological properties include raised intraocular pressure (IOP) [1]. The mechanism by which it increased IOP has also attracted much comments and reports. It is still not completely understood. For a long time, it was accepted that contraction of the extraocular muscles (during fasciculations) causes compression of the globe and raises IOP. This idea became doubtful after the work of Kelly et al. [2]. They measured intraocular pressure in a series of patients whose extraocular muscles had been unilaterally severed before elective enucleation and compared the pressure changes with those of the contralateral eye, with the extraocular muscles intact. The authors noticed that there were no differences in intraocular pressure between the eyes, before and after intravenous injection of suxamethonium. There was significant increase in IOP in both eyes to about the same extent.

This observation by Kelly et al. [2] stimulated the proposition of other hypotheses to explain IOP increase after suxamethonium. Kelly et al. [2] postulated that the most likely mechanism of raised IOP by suxamethonium is its effect on aqueous humour fluid dynamics. They relied on previous study that showed that suxamethonium has cycloplegic effects on the eye, causing relaxation of accommodation and reduction of axial thickening of the lens [2]. The time course of this cycloplegia parallels the metabolism of suxamethonium by pseudocholinesterase as well as that of raised IOP [2].

Due to the action of suxamethonium to raise IOP in the intact eye, its use in penetrating eye injuries and open globe surgery has been very contentious. Rational reasoning and literature reports [3, 4] suggest that ocular contents, especially vitreous humour, could be expelled from the eye in such situations. This may cause permanent blindness in the patient. However, many competent authorities dispute these reasoning and reports [5] and argue that suxamethonium can be safely used in well-anaesthetised patients with open globe [5, 6]. The anaesthetist is thus faced with the dilemma of what to do when rapid sequence induction of anaesthesia is desired in full-stomach patients, with penetrating eye injuries, billed for emergency surgery. Alternatives to suxamethonium in such instance do not exactly replicate the actions of suxamethonium in rapid onset and offset of action. For example, whereas large-dose rocuronium (1.2 mg/kg) approximates suxamethonium in onset of action and intubating conditions, suxamethonium is considered clinically superior for its shorter duration of action [7]. Reports that seem to implicate suxamethonium in extruding ocular contents in the anaesthetized patient are all anecdotal [3, 4, 8]. There is as yet, no formal, well-documented case report of this phenomenon in the medical literature, to our knowledge. We present this case report of the inadvertent loss of vitreous humour in a polytraumatized, road traffic accident patient, for emergency laparotomy and right corneal repair after a midazolam-ketamine-suxamethonium induction of anaesthesia.

## 2. Case Presentation

A 34-year-old man was brought to the Accident and Emergency Unit of the University of Benin Teaching Hospital, Benin City, Nigeria. The patient was a victim of road traffic accident about 2 hours before presentation. Clinical examination showed an anxious-looking man, with multiple bruises on the face, anterior abdominal wall, and right upper arm. He was clinically pale, with a pulse rate of 122 bpm, and blood pressure of 90/60 mmHg. The respiratory rate was 32/min, but the chest was clinically clear. The abdomen was distended globally tender with guarding and absent bowel sounds. Abdominal paracentesis revealed hemoperitoneum. Ophthalmological examination showed right corneal laceration with intact intraocular contents.

Results of preoperative investigations were packed cell volume (PCV) of 22%, normal serum electrolyte and urea levels, and normal urinalysis. Four units of whole blood were cross-matched. A diagnosis of intra-abdominal visceral damage with right corneal laceration, in a polytraumatized patient, was made. A decision to perform an emergency laparotomy before corneal repair was made. The damaged right eye was strapped with sterile gauze. The patient was resuscitated with 2 litres of normal saline and 500 mL of isoplasma, given intravenously. He was given intravenous metoclopramide 10 mg and ranitidine 50 mg.

In the operating theatre, monitors were attached. Baseline pulse rate was 100 bpm, and blood pressure was 110/70 mmHg. SpO_2_ was 96–99% in room air. Anaesthesia was induced with intravenous midazolam 10 mg, ketamine 100 mg, while cricoid pressure was applied on loss of consciousness. Intravenous suxamethonium 100 mg was given Laryngoscopy and endotracheal intubation were done after the fasciculations. The blood pressure rose to 128/85 mmHg after endotracheal intubation and returned to baseline levels after about six minutes. The patient was connected to the anaesthetic machine via a circle absorber breathing system. He was mechanically ventilated with 100% O_2_ and 1-2% halothane. Correct placement of the endotracheal tube was confirmed by chest auscultation and observation of the pulse oximeter. Intravenous fentanyl 100 *μ*g was given, followed by 6 mg pancuronium i.v. on return of spontaneous breathing. Anaesthesia went well, with vital signs within normal limits.

The surgical findings at laparotomy were ruptured spleen and transverse colon. Splenectomy and colon repair were done. Thereafter, the ophthalmologists were invited to come and effect the right corneal repair, while the patient was still under anaesthesia. The sterile gauze dressing was removed. On examining the eye, it was found that the vitreous humour has been extruded. They decided to perform evisceration on a later date. The eye was redressed.

Anaesthesia was terminated and the patient was ventilated with pure oxygen. Muscle paralysis was reversed with neostigmine 2.5 mg i.v. and atropine 1.2 mg i.v. The airway was suctioned and the patient was extubated on return of spontaneous breathing. He was transferred to the Post-Anaesthesia Care Unit (PACU). After about 45 minutes, the patient was transferred to the ward, with full consciousness and stable vital signs. 

## 3. Discussion

The cause of vitreous humour extrusion in this patient, based on the concept of preponderance of probability, is most likely due to suxamethonium. Other more remote causes could be the use of ketamine at induction of anaesthesia and the pressure of the face mask on the right globe during anaesthetic preoxygenation of the patient. The report of the effect of ketamine on intraocular pressure in the literature is conflicting. While some reports claim it increased IOP [9], others claim drop in IOP [10], and yet others claim no effect [10]. Studies that showed increased IOP with ketamine used doses far in excess of what is used in clinical practice [10]. Thus, it can safely be stated that clinical doses of ketamine (<3 mg/kg i.v.) do not increase IOP. It is therefore unlikely that the induction dose of ketamine (100 mg) in this 74 kg man contributed to the vitreous humour extrusion. The probability that the pressure of the face mask on the globe is responsible is very unlikely, as extreme care was taken to avoid this during anaesthetic preoxygenation of the patient. This thus leaves suxamethonium as the only confounding variable to explain the inadvertent vitreous extrusion in this patient.

There are theoretically other factors that could raise IOP and probably cause or contribute to the vitreous extrusion. These include inadequate depth of anaesthesia before intubation, the hypertensive response to intubation, carbon dioxide (CO_2_) retention from suxamethonium apnoea, bucking, and straining from inadequate neuromuscular blockade. We do not think any of these apply to our patient. Steps were taken to ensure sufficient depth of anaesthesia at induction. Hence, we used a combination of intravenous midazolam (10 mg) and ketamine (100 mg). Each of these drugs at the dose used can induce anaesthesia on its own. In addition, midazolam reduces intraocular pressure [11]. Deep anaesthesia by itself, mitigates the hypertensive response to intubation and rises in IOP [12]. In this patient, there was a modest rise in blood pressure after intubation from the baseline value of 110/70 mmHg to 128/85 mmHg after intubation. This rise is not clinically significant. CO_2_ retention and inadequate neuromuscular blockade are unlikely, given the fact that suxamethonium is still the fastest neuromuscular blocking agent with excellent relaxation. Duration of apnoea is thus minimal, with no bucking or straining at intubation with appropriate dose.

 Lincoff et al. [3], while reporting their study of the effects of suxamethonium on IOP, reported an anecdotal personal communication from surgical colleagues (ophthalmologists) and stated interalia: “since the publication of the previous article (describing the effects of succinylcholine on IOP), various communications have been received from ophthalmologists who used succinylcholine at surgery. These included several reports of cases in which succinylcholine was given to forestall impending vitreous prolapse, only to have a prompt expulsion of vitreous occur” [4]. Four instances of such personal communication were given, with no further details. In the same year that Lincoff's report appeared (1957), Dillon et al. [4] reported another anecdotal personal communication from an ophthalmologist colleague. In their report, they stated interalia: “it has been reported to us by Godman that a small amount of vitreous was lost from the eye of a patient undergoing cataract surgery wherein succinylcholine was administered to the patient under light anaesthesia at the time that the sclera had been incised and the anterior chamber opened” [4]. Dillon et al. [4] actually went on to conclude that “it would appear, therefore, that the administration of succinylcholine for intraocular surgery is at least hazardous and possibly contraindicated.”

 The anecdotal reports of Lincoff et al. [3] and Dillon et al. [4] were very instrumental in forging since their days, a near unanimous clinical opinion that suxamethonium is contraindicated in penetrating eye injury or open eye surgery. When Libonati et al. [5] reported in a retrospective study that there was no extrusion of ocular contents with the use of suxamethonium in penetrating eye injuries, there was a spate of discussion and letters to the editor. One of these letters contained another anecdotal report of the loss of ocular content with the use of suxamethonium in penetrating eye injury [8]. Rich et al. [8] reported thus: “the expulsion of intraocular content after succinylcholine induction is more than merely a theoretical concern. One of us (A. L. R.) has witnessed this complication, and the result was enucleation following a simple scleral laceration” [8].

Chidiac [12] reported a retrospective study at their institution, where suxamethonium was used in 8 cases of open eye surgery. There were no reports of vitreous loss, no lens or uvea extrusion, and no excessive intraocular bleeding. Chidiac, however, added that suxamethonium administration was preceded with drugs that attenuate its intraocular pressure effects, such as thiopentone, propofol, narcotics, nifedipine, or lignocaine [12]. Chidiac's paper [12], together with many others before and after it, suggests that suxamethonium may be used safely in open eye surgery after steps are taken to mitigate its tendency to raise IOP. These steps include pretreatment with nondepolarizing muscle relaxants and good depth of anaesthesia before suxamethonium administration. Many competent authorities believe that pretreatment with nondepolarizing muscle relaxants has no effect on suxamethonium-induced rise in IOP [13].

Other pharmacologic agents that reduce IOP can obtund the intraocular hypertension caused by suxamethonium. These include intravenous opioids like fentanyl, alfentanil [14], and alpha-2 agonist like dexmedetomidine [15]. While these agents may not prevent the rise in IOP with suxamethonium and intubation, the rise does not get beyond baseline values with alfentanil [15] and dexmedetomidine [15]. In retrospect, we believe that the vitreous extrusion in this patient may have been prevented if further steps were taken to deepen anaesthesia, by administering the fentanyl before giving the suxamethonium. This would have further reduced IOP (in addition to the effect of midazolam) and may be completely mitigate the IOP rise with suxamethonium and endotracheal intubation.

In these days of evidence-based practice, perhaps only large-dose rocuronium (1.2 mg/kg) rivals suxamethonium in fast-onset of action [7] and thus offers a suitable alternative for rapid-sequence induction technique. It, however, has a longer duration of action. In situations of “cant intubate, cant ventilate,” sugammadex should be handy for rapid termination of muscle paralysis. Where this is not available, suxamethonium may still be the best option.

## 4. Conclusion

The place of suxamethonium in open eye surgery has both proponents and opponents. It is pertinent to note that the misgivings about suxamethonium for open eye surgery have arisen from anecdotal reports, of which only 3 have been reported from 1957 to the time of writing this paper [3, 4, 8]. These anecdotal reports are not formal peer-reviewed case reports, but rather personal communications. On the other hand, several retrospective studies in humans [5, 12] suggest that suxamethonium can safely be used in open eye surgery in the well-anaesthetized patient, without extrusion of ocular contents. It is further argued that anecdotal reports are not sufficient to sustain the teaching against the use of suxamethonium in penetrating eye injury or open globe surgery. However, our own experience with this patient has taught us that the dictum, primum nonnocere, is still very relevant with the use of suxamethonium in the open globe. 
